# Non-syndromic Intellectual Disability: An Experimental In-Depth Exploration of Inheritance Pattern, Phenotypic Presentation, and Genomic Composition

**DOI:** 10.7759/cureus.34085

**Published:** 2023-01-23

**Authors:** Qaisar Ali Khan, Rohail Khan, Ravina Verma, Suchi D Shah, Bhavana Vattikuti, Aleena Z Khan, Andia Shahzadi, Parsa Abdi, Michelle Anthony, Christopher S Farkouh, Matthew Farkouh, Natalia Santiago, David Zepeda, Andrew Nunez

**Affiliations:** 1 Internal Medicine, Medical Teaching Institution-Khyber Teaching Hospital (MTI-KTH), Peshawar, PAK; 2 General Medicine, Shifa College of Medicine, Islamabad, PAK; 3 General Medicine, St. George's University, West Indies, GRD; 4 Internal Medicine, Ahmedabad Municipal Corporation Medical Education Trust (AMC MET) Medical College, Ahmedabad, IND; 5 Internal Medicine, Cebu Doctors' University College of Medicine, Cebu City, PHL; 6 General Medicine, Islamabad Medical and Dental College, Islamabad, PAK; 7 Neurosurgery, Lenox Hill Hospital/Donald and Barbara Zucker School of Medicine, New York City, USA; 8 Medicine, Memorial University of Newfoundland, St. John's, CAN; 9 Pathology, University of Arizona College of Medicine, Tucson, USA; 10 General Medicine, Rush Medical College, Chicago, USA; 11 Emergency Medicine, Ponce Health Sciences University, Ponce, PRI; 12 General Medicine, Universidad Autónoma de Guadalajara School of Medicine, Guadalajara, MEX

**Keywords:** autosomal recessive, candidate genes, genetic analysis, intellectual disability, mental health

## Abstract

Background

Intellectual disability (ID), also termed mental retardation (MR), is a neurodevelopmental disorder characterized by an intelligence quotient (IQ) of 70 or below and a deficit in at least two behaviors associated with adaptive functioning. The condition is further classified into syndromic intellectual disability (S-ID) and non-syndromic intellectual disability (NS-ID). This study highlights the genes associated with NS-ID.

Objectives

A genetic study was performed on two Pakistani families to know the inheritance patterns, clinical phenotypes, and molecular genetics of affected individuals with NS-ID.

Methodology

Samples were collected from two families: families A and B. All affected individuals in both families were diagnosed by a neurologist. Written informed consent was taken from the affected individuals and guardians before collecting the data and sample. Family A belongs to the Swabi District of Pakistan having four affected individuals, out of whom three were male and one was female. Family B also belongs to the Swabi District of Pakistan having two affected individuals, out of whom one was male and one was female. A total of 10 candidate genes were selected and were further screened by microarray analysis.

Results

In family A, this analysis identified a region of 9.6 Mb on chromosome 17q11.2-q12 between the single nucleotide polymorphisms (SNPs) rs953527 and rs2680398. The region was genotyped using microsatellite markers to confirm the haplotypes in all family members. Based on the phenotype-genotype relationship, 10 possible candidate genes were selected out of more than 140 genes in this critical region of 9.6 Mb. In family B, homozygosity mapping through microarray identified four homozygous areas of affected individuals: two (27,324,822-59,122,062 and 96,423,252123,656,241) on chromosome 8, one (14,785,224-19,722,760) on chromosome 9, and one (126173647-126215644) on chromosome 11.

Conclusion

An autosomal recessive pattern was found in the pedigrees of both families A and B. Phenotypically affected individuals showed IQ levels below 70. Three genes, CDK5R1, OMG, and EV12A, were found on chromosome 17q11.2-q12 region of affected individuals in family A with high expression in the frontal cortex of the brain, hippocampus, and spinal cord, respectively. Other regions on chromosomes 8, 9, and 11 as evident from the affected individuals in family B can also contribute to the non-syndromic autosomal recessive intellectual disability (NS-ARID). Further research is needed to find the association of these genes with intelligence and other neuropsychiatric conditions.

## Introduction

Intellectual disability (ID) is a neurological condition characterized by an impairment of intellectual capacity and adaptive behavior diagnosed at the age of 18 years [1]. The prevalence of ID ranges from 1% to 3% in all populations, and the prevalence of severe ID and mild ID is variable due to the influence of some environmental stressors [2,3]. Studies have shown that non-syndromic intellectual disability (NS-ID) is extremely heterogeneous and contributes much more than X-linked ID [4]. The Diagnostic and Statistical Manual of Mental Disorders (DSM) classified the severity level of ID based on IQ level and daily skills into four categories: mild, moderate, severe, and profound [5]. In NS-ID, intellectual disability is the main clinical feature. It has been difficult to rule out the associated subtle neurological and psychiatric conditions, making the diagnosis difficult [4].

Environmental and genetic factors can contribute to most IDs, but for most cases, the exact cause is still unknown [6]. Genetic causes account for about 25%-50% of ID cases, but this number is increasing proportionally with severity. Other causes include chromosomal abnormalities, autosomal trisomies, and copy number variants (CNVs) [4,7]. Previously, single genes causing NS-ID have been identified, and it has been evident that these genes also interact with other neurodevelopmental phenotypes, thus making the etiology multifactorial with more than one gene contributing to the disease [4]. Due to the innate heterogeneity of the NS-ID phenotypes, genetic analysis of the condition is also complex. However, different techniques such as homozygosity mapping and microarrays are used to identify the genes and their loci [4]. This study aimed to identify the inheritance patterns, clinical phenotypes, and molecular genetics of two Pakistani families having affected individuals of NS-ID. The sub-objectives of this work include genotyping of reported loci/genes, mutations in selected genes, and bioinformatics analysis to resolve protein models and their protein interactions with others.

## Materials and methods

Two families, family A and family B, were included in this study. Both families have physician-diagnosed NS-ID individuals. A standard method was used to draw the pedigree of each family, and detailed information about each family was recorded on the study-designed Performa [8]. Peripheral blood (5 mL) was sampled using the venipuncture technique with a sterile syringe and ethylenediaminetetraacetic acid (EDTA) vacutainer for everyone. Samples were kept at 4°C in the laboratory refrigerator until genomic DNA extraction. The standard phenol-chloroform method (also known as the organic method) was applied for the extraction and purification of the genomic deoxyribonucleic acid (DNA) from the collected blood samples. A flask containing 40 mL of 1X tris-borate-EDTA (TBE) buffer (89 mM tris-borate and 2.5 mM EDTA, pH 8.3) and 0.6 g of agarose was filled with 2 μL of an ethidium bromide solution (0.1 ug/mL), making it easier to visualize DNA by electrophoresis. After mixing, the ingredients were poured into a gel caster. The gel solidified at room temperature after 20-25 minutes. DNA samples (5 μL) were loaded on 1.2% agarose gel with 3 μL bromophenol blue dye. In a 1X TBE buffer, electrophoresis was carried out for 20 minutes at 120 V (400 mA), and the gel was observed under Dolphin-Doc (Wealtec Corporation, Sparks, NV, USA) to enhance the results. PCR was used to amplify microsatellite markers. The markers were visualized by a UV trans-illuminator gel stained with ethidium bromide. The repeat polymorphism and heterozygosity of the markers were used as a selection tool. The cytogenetic positions of markers were collected from a map created by Rutgers University [9]. A total of 11 genes that are involved in neuronal development and showed association with NS-ID were selected for conducting this study as shown in Table 1.

**Table 1:** Genes selected for conducting this study OMIM: Online Mendelian Inheritance in Man; ID, intellectual disability; NS-ARID, non-syndromic autosomal recessive intellectual disability; NS-ARMR, non-syndromic autosomal recessive mental retardation

Genes	OMIM number	Location	Function
PRSS12	606709	4q26	Encodes the gene for the neuronal serine protease neurotrypsin, which is connected to brain development and may be a cause of NS-ARID [9].
CRBN	609262	3p26.2	Encodes the cereblon protein, which contributes to the development of the brain and may be connected to NS-ARID [10].
CC2D1A	610055	19p13.12	All NS-ARID patients have a stop codon at position 438 of the mutant protein, which encodes the coiled-coil and CC2D1A protein [11].
GRIK2	138244	6q16.3	Encodes a kainate receptor subunit that is highly expressed in the brain and is involved in synaptic transmission. The GRIK2 gene in the afflicted NS-ARID people had a complicated mutation [12].
TUSC3	601385	8p22	Encodes a 348-amino acid protein that appears to be involved in the NS-ARMR and in catalyzing the key step in the N-linked protein glycosylation pathway that is implicated in congenital abnormalities of glycosylation [3].
TRAPPC9	611966	8q24.3	Tremendously expressed and connected to NS-ARID in the post-mitotic neurons of the cerebral cortex [13,14].
TECR	610057	19p13.12	ID families have been linked to a homozygous C to T transition in exon 8 of the TECR gene that causes the missense mutation P182L [15].
ST3GAL3	606494	1p34.1	Two families with NS-ARID were found to have missense mutations in the gene that encodes beta-galactoside alpha-2,3-sialyltransferase II, a Golgi membrane protein, and two unrelated mutations [16].
MED23	605042	6q23.2	A crucial factor controlling the expression of protein-coding genes is a mediator complex component. A family with five members who had the NS-ARMR mutation was reported to have a homozygous missense mutation [17].
MAN1B1	604346	9q34.3	It contains the gene for the enzyme endoplasmic reticulum mannosyl-oligosaccharide 1,2-alpha-mannosidase, which is essential for the maturation of N-linked glycans in the secretory route. Families with NS-ARID were shown to have mutations [18].
FBXO31	609102	16q12.2q21	Found to be expressed in the cell body of primary hippocampal neurons from E15 mouse embryos and was found in families with ID [19].

Linkage to candidate gene loci

The database of Genotypes and Phenotypes (dbGaP), database of Single Nucleotide Polymorphisms (dbSNP), GenBank, and Genome Data Viewer (GDV) were explored, and the linkage analysis technique was used to find the loci of the genes and their association with NS-ID. The sample from both families was initially evaluated for the already known genes causing NS-ID. The microsatellite markers used to genotype members of families A and B are shown in Table 2.

**Table 2:** Microsatellite markers used for the linkage analysis

Gene	Markers	Forward	Reverse
TRAPP C9	D8S256	GTTCAAGGGCTCAGGGTTCT	CTTCCACCTTTAGCCAAGGA
	D8S1204	CCACTCTCATCTACCACCAC	TACCCCTTCTCCCAACTTAC
	D8S1743	TGAATACTTAAAGTTTGCTA AAAGG	TGAGGTCTGCTGCTGG
	D8S1837	AATGAAAGGCTGACCTCC	ACCCAGATTGCTTATGCTC
FBXO3 1	D16S305	CCTCCCAGGTTCAGGCAATT CTTCT	TAGGCGACAGAGTGGGACT CCATTA
	D16S402	TTTTGTAACCATGTACCCCC	ATTTATAGGGCCATGACCA G
	D16S520	GCTTAGTCATACGAGCGG	TCCACAGCCATGTAAACC
	D16S262 1	GTCATATGGGCCAATTCCC	TACCGCGTAGTGAGACTGT G
TUSC3	D5S261	TGCCACTGTCTTGAAAATCC	TATGGCCCAGCAATGTGTAT
	D5S549	TCTGATTAGCCAACCTCTAA CA	CTTACAAACACCACTATGC ATG
	D5S1731	CCAAGCAAATCATGGAAAT C	AGCAAACTTATCCCACAAG G
	D5S1827	GACAGAATCATGTGGCCTTT	TTTTGTAAAATGTAAAATTG GCTTT
PRSS12	D4S191	GATCATAGCTTCCTCTCTTT GAATA	AAAATGAGCCCACTAGAGA TGGTA
	D4S2392	CAGCCTTCCAAATAGCTTGA	TAGTAAGCAGCTCATTTTGG C
	D4S3024	CTGGAAGCCAGGTAGGA	AACACTTAGAACTTGCAGC C
NSUN2	D5S406	AAACATACCTCTTCCCACAC A	GATGCTAACTGCTGACTATG C
	D5S580	TAGTCTCTTCATGACTTGGT A	CTGCATTCTAGCCTGGGC
	D5S630	CTCTTCGCTCTTCTTTCTCC	TCACTGCTTTACCTTTCAGT G
	D5S1981	CCTGTACCAATCCATGC	GAGCCATGTGAGTGTCC
	D5S2505	TGTTGGAAGACTTCTCAGCC	CACACATGCTGTGTCTCTCA
CRBN	D3S3525	GATTTTGGGACTTGTCAGC	GTGTAATCTCCTGTAGCAGA GTTC
	D3S1560	GCATCTACAGGGGGTGTCT	AGGCTGATTTTCAGCACAA
	D3S2777	CCCATCAATGACATGCTACC	CGGCAGTCTAGAATACCAG
CC2D1 A	D19S840	ATAGGCCAAGACTGTCTAA AACAA	GCCCTAACTGCTGTAAGAG AACT
GRIK2	D6S1543	CTGAGATAGAGTAGTATCA CCAGCG	GTGGAACATAAAGCAGCCC
	D6S434	CAGGTAGTCCCCCAAAATC A	AGCTCAGGGCTTATGCCAG T
	D6S283	GGTTTTACAAAATCCTGTCC TGCTT	CTATAGTGCTGATTTCTCTC TCTC
ST3GA L3	D1S211	AGCTACATGGCAGGATCAG A	GGATTCCTTGCTCTGGAAAG
	D1S353	TATTGCTTCCATGGGGGT	TTGGGTTCTGCCTTTAGC

The Affymetrix GeneChip® Human Mapping 10K Array was used to undertake genome-wide linkage analysis in family members (version 2.0) [20]. The 10K Chip-Array in this edition has 10,204 SNPs with a mean inter-marker distance of 258 kb or 0.36 cM. The data for SNPs with Mendelian errors rate of more than 4% were removed by programmed Ped Check. The program MERLIN was used to detect non-Mendelian errors and perform nonparametric linkage analysis and pedigree analysis on all chromosome genotypes simultaneously [11]. A modified version of the programmed ALLEGRO was used to perform the metric linkage analysis through the stepwise use of a sliding window with sets of 150 or 300 SNPs [21].

Haplotypes were reconstructed with ALLEGRO and presented graphically with Haplopainter. This program also reveals informative SNP markers as points of recombination between parental haplotypes. To handle all data, the graphical user interface ALOHOMORA was used to facilitate linkage analysis with chip data [12]. A maximum logarithm of the odds (LOD) score of 3.26 was obtained from family A for a single region located on chromosome 17q11.2-q12 between SNPs rs953527 and rs2680398, defining a shared critical interval of about 9.6 Mb. Subsequent fine mapping using microsatellite markers confirmed homozygous haplotypes for the linked region in all affected individuals. Of the 142 protein-coding genes located within the critical region (UCSC Genome Browser), we selected 10 highly relevant functional candidate genes, i.e., ACCN1 (MIM 601784), CDK5R1 (MIM 603460), EVI2A (MIM 158380), EVI2B (MIM 158381), MYO1D (MIM 606539), OMG (MIM 164345), PEX12 (MIM 601758), RAB11FIP4 (MIM 611999), RHBDL3, ZNF207 (MIM 603428), and the two miRNA genes MIR193A and MIR365-2 based on expression in the brain, involvement in neurodevelopmental pathways, and association with NS-ID for direct sequence analysis.

Consent and ethical approval

All participants of the study were informed of the purpose of this study prior to their consent to participate. Written informed consent was obtained from all participants, including the affected individuals, their parents, and other normal members of the affected families. The study protocol conforms to the ethical guidelines of the 1975 Declaration of Helsinki. Ethical approval has been granted by the institutional review board of District Headquarter (DHQ) and Teaching Hospital, Kohat Development Authority (KDA), Kohat, Khyber Pakhtunkhwa, Pakistan, with reference number 3224.

## Results

Family description and pedigree analysis

Family A

**Figure 1:**
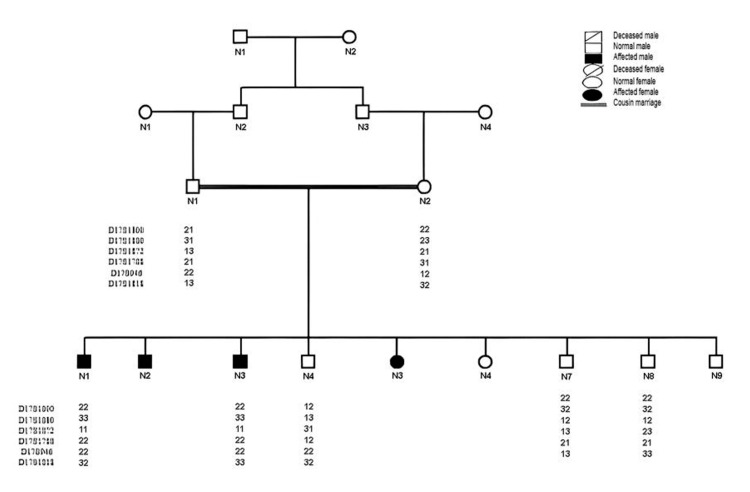
Detailed pedigree of family A

Family A (consisting of four individuals) belonged to the Swabi District of Khyber Pakhtunkhwa. Autosomal recessive inheritance was found throughout the pedigree. Compared to his more affected siblings (IV-2, IV-3, and IV-5), the 25-year-old male (IV-1) showed moderate ID. There were no other neurological symptoms such as ataxia or epilepsy in the affected family members nor any congenital disease of other organ systems. All affected individuals had average head circumferences and could not learn to read or write. All affected family members’ speech development was delayed, but their motor development was normal, except for slight cerebellar atrophy. Magnetic resonance imaging (MRI) revealed no significant anatomical defects in IV-1 and no evidence of typical facial dysmorphism. Standard blood biochemistry testing also proved normal. Additionally, both parents were noted to be in good mental health. The detailed pedigree of family A is shown in Figure 1.

Family B

**Figure 2:**
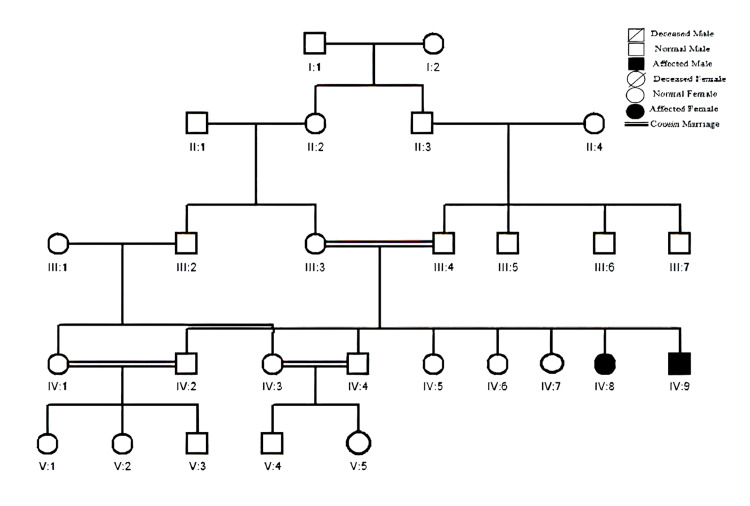
Detailed pedigree of family B

Family B belonged to the Swabi District of Khyber Pakhtunkhwa region of Pakistan. A detailed pedigree revealed a recessive inheritance pattern. The family provided five peripheral blood samples, three of which were normal (III:3, III:4, and IV:5) and two of which were affected (IV:8 and IV:9). The affected individuals in the sample, IV:8 and IV:9, were 38 and 24 years old, respectively. One of the affected individuals (IV:8) had a seven-year delay in walking, poor memory, and hearing difficulty. Ectodermal dysplasia, poor memory, counting difficulties, and obesity were seen in the other affected individual (IV:9). The detailed pedigree of family B is shown in Figure 2.

Gene expression analysis

Family A

**Figure 3:**
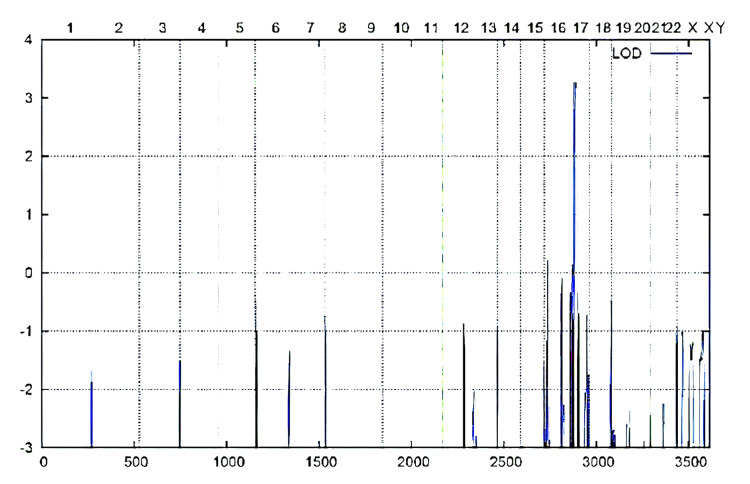
Illustration of genome-wide LOD score calculations following 10K array SNP genotyping ALLEGRO LOD scores are given along the y-axis relative to the genomic position in cM on the x-axis. The highest peak was noted on chromosome 17q in the telomere region. LOD: logarithm of the odds

**Figure 4:**
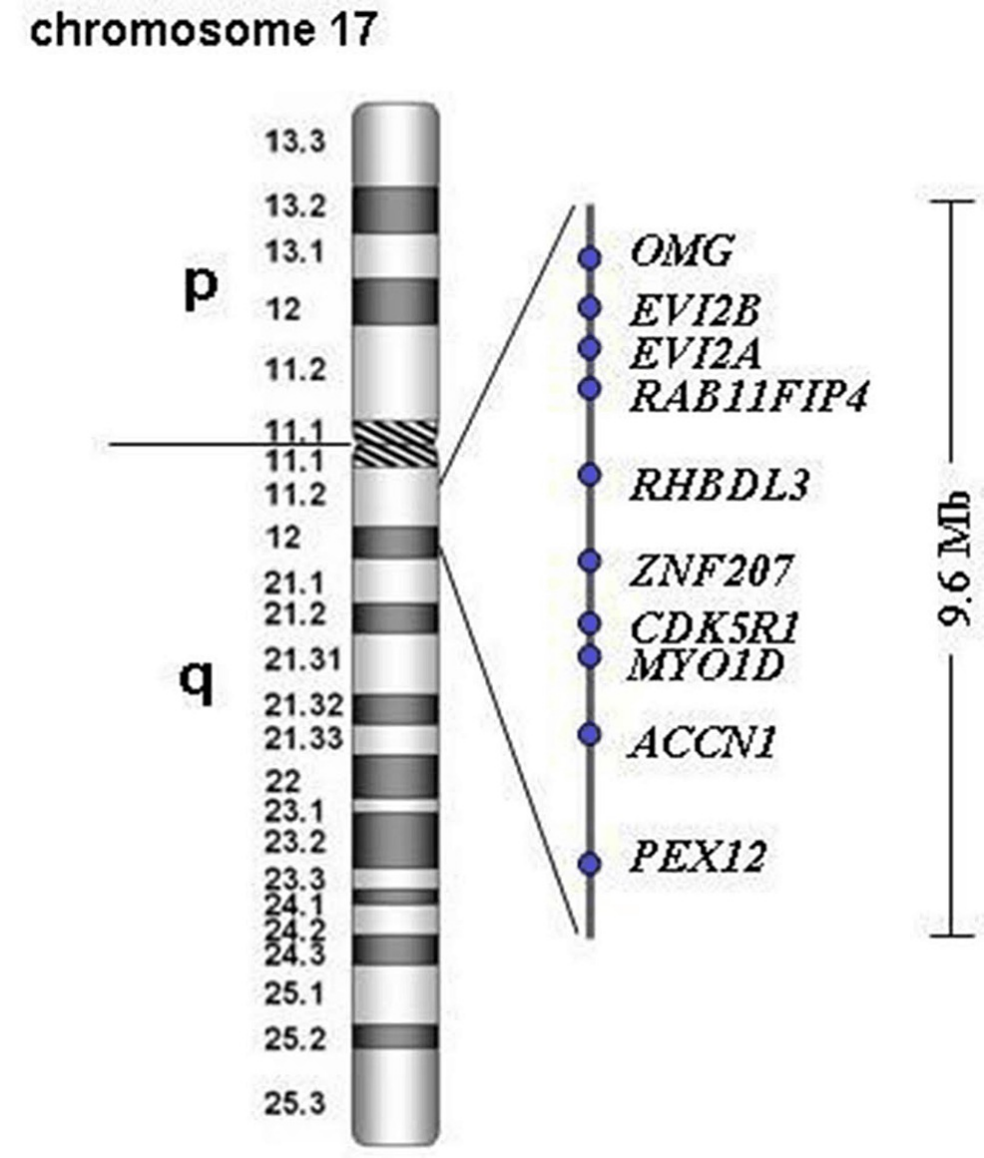
Genomic overview of the chromosome 17q11.2-q12 region and genomic localization of the analyzed candidate genes located within the critical region spanning 9.6 Mb

The results suggested 10 possibly relevant functional candidate genes in the region with a high LOD score. Further sequencing of these genes is required to confirm the mutation. Genome-wide LOD score was calculated following 10K array SNP genotyping. The highest peak was noted on chromosome 17q in the telomere region as shown in Figure 3 and Figure 4.

**Figure 5:**
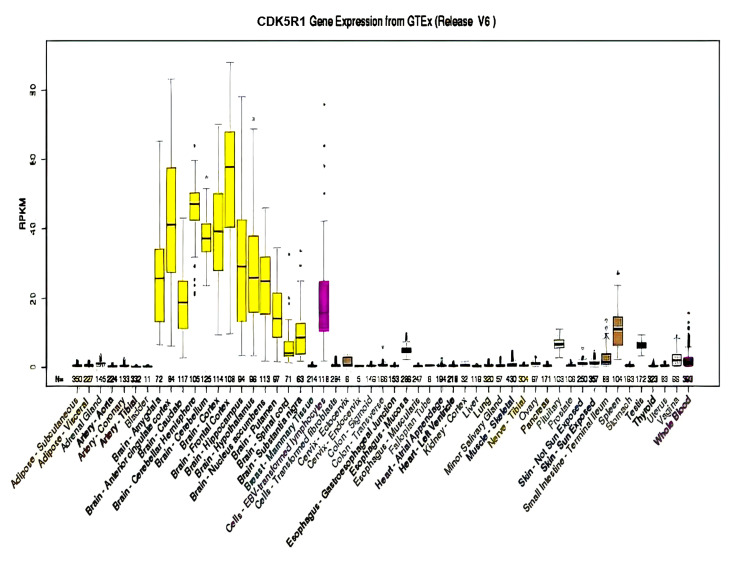
Expression of CDK5R1 in different organs of the human body

**Figure 6:**
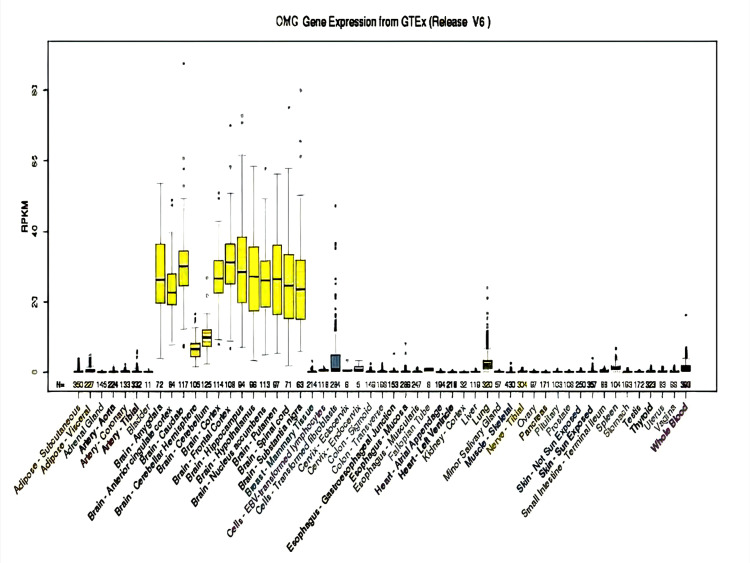
Expression of OMG in different organs of the human body

**Figure 7:**
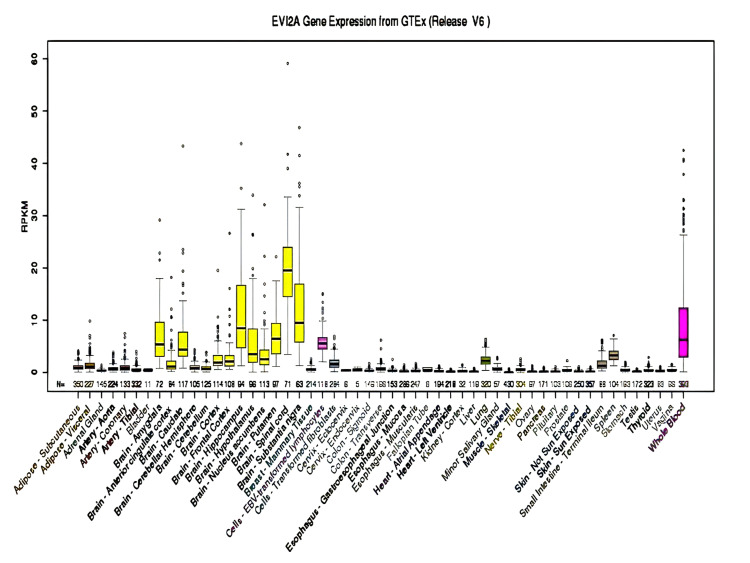
Expression of EVI2A in different organs of the human body

Three of these 10 genes (CDK5R1, OMG, and EVI2A) show higher expression in the brain, especially in the cerebral cortex, which makes the possibility of these genes the candidate genes for NS-ID. The expression CDK5R1, OMG, and EV12A genes from GTEx in various organs of the human body are shown in Figure 5, Figure 6, and Figure 7, respectively.

Family B

**Figure 8:**
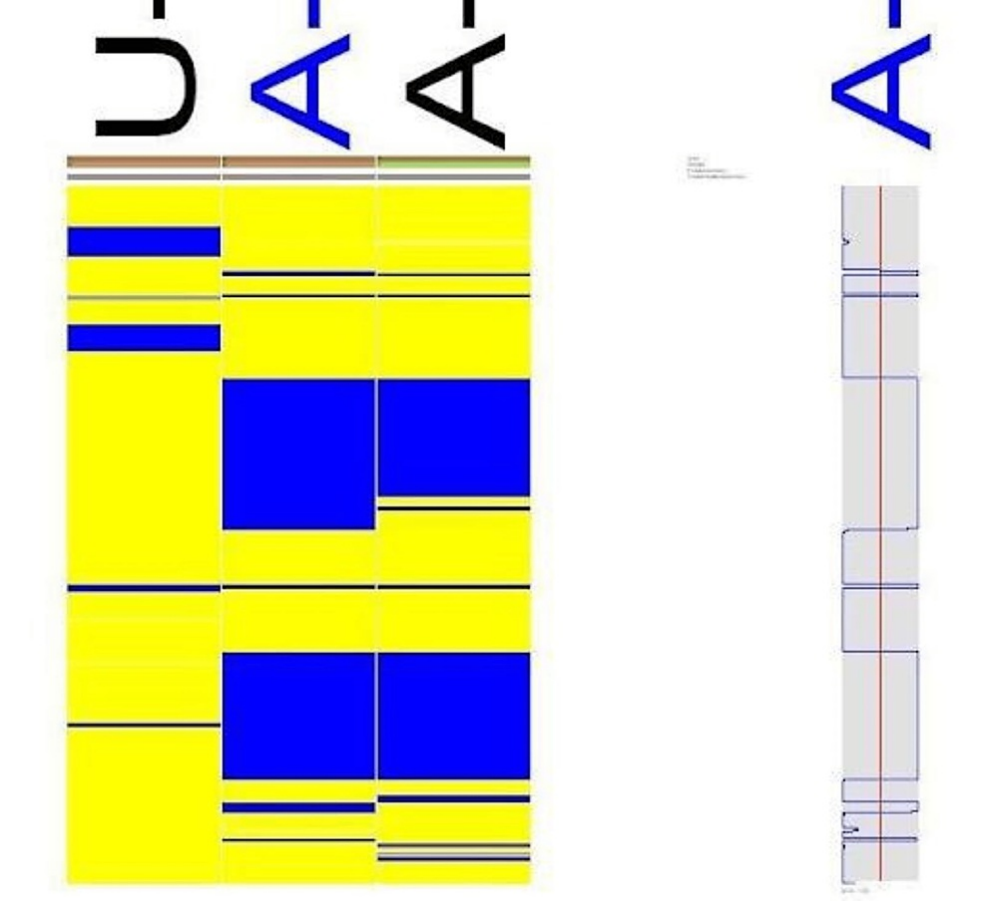
Microarray results of family B chromosome 8 (27,324,82259,122,062)

**Figure 9:**
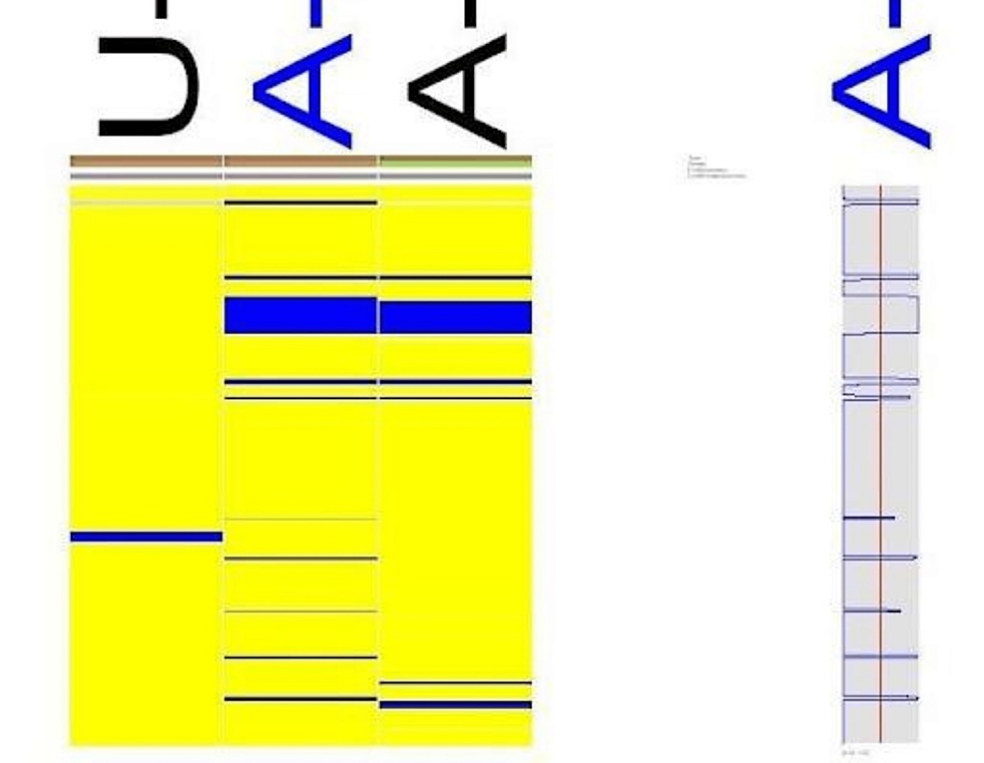
Microarray results of family B chromosome 9 (14,785,224-19,722,760)

**Figure 10:**
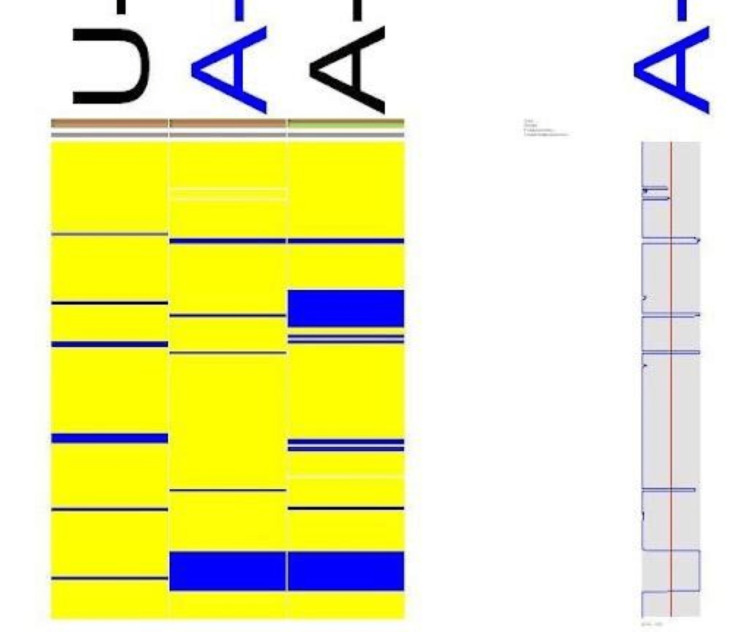
Microarray results of family B chromosome 11 (126173647-126215644)

Microarray on three samples of family B was carried out, and the results showed two homozygous regions of the affected individuals in chromosome 8, i.e., 27,324,82259,122,062 and 96,423,252-123,656,241; one homozygous region in chromosome 9, i.e., 14,785,224-19,722,760; and one region in chromosome 1, i.e., 126173647-126215644, as shown in Figure 8, Figure 9, and Figure 10, respectively.

## Discussion

The pedigree analysis showed that the inheritance pattern in both families was autosomal recessive; it was more in consanguineous marriages. In both pedigrees of family A and B, the parents were asymptomatic. All included participants from both families had an IQ level of less than 70 and did not show syndromic features, except the IV:9 who had ectodermal dysplasia, poor memory, counting difficulties, and obesity and had been excluded from further genetic studies. In family A, the affected individual was identified as sharing a critical region of 9.6 Mb between rs953527 and rs2680398 on chromosome 17q11.2-q12. Fine mapping using microsatellite markers confirmed homozygous haplotypes for the linked region in all affected individuals. We selected 10 possible candidate genes, namely, ACCN1, CDK5R1, EVI2A, EVI2B, MYO1D, OMG, PEX12, RAB11FIP4, RHBDL3, and ZNF207, out of approximately 150 genes lying in this critical region of 9.6 Mb. These selections were based on expression in the brain, their role in neurodevelopmental pathways, and their association with NS-ID. We found that three out of the 10 genes (CDK5R1, OMG, and EVI2A) have relatively higher expression in different brain parts. Mutation screening of candidate genes will identify the variants as the causative agent in the presented family. Homozygosity mapping through microarray identified four homozygous regions in affected individuals: two at chromosome 8 (27,324,82259,122,062 and 96,423,252-123,656,241), one at chromosome 9 (14,785,224-19,722,760), and one at chromosome 11 (126173647-126215644). Three essential genes in region 27,324,822-59,122,062 of chromosome 8 include ADAM18, SFRP1, and NKX6-3. ADAM18 and NKX6-3 are involved in neurogenesis and CNS development, respectively; therefore, they may be involved in the pathogenesis of ID in family B.

Different methods including microarray technologies and homozygosity mapping are applied nowadays to identify the causative genes and associated chromosomal abnormalities [4]. So far, microarray in combination with homozygosity mapping has been used for the identification of all genes associated with autosomal recessive NS-ID in large consanguineous families. This is a fast-screening method and helps in the identification of the homozygous region of the genome for the same alleles [4]. The main drawback of homozygosity mapping is that it identifies a large region on the chromosome in which disease-relevant candidate genes are located. The selection of candidate genes from such a large region is difficult, and next-generation sequencing (NGS) is used to overcome this problem [4,22]. Until now, only six NS-ID genes have been reported, and only two (TUSC3 and TRAPPC9) among these have been identified in multiple families. TUSC3 encodes a protein involved in N-linked protein glycosylation [23,24]. TRAPPC9 encodes a protein called NIBP and is involved in the activation of the nuclear factor-κB (NF-κB) pathway [25]. It has been shown that TRAPPC9 is involved in axonal growth in vitro and involvement in neuronal cell survival [25]. It has been found that the rs10119 variant in the APOE/TOMM40 area has a more substantial adverse effect on cognitive performance with increasing age, indicating that various genetic variables explain cognitive ability in old age [26].

The limitations of this study include the modest sample size and the selection of a small number of candidate genes. Also, this study did not highlight the gene relationship in terms of other phenotypic traits such as height and body mass index. Further studies are needed to know the association of NS-ARID genes with normal learning, intelligence, and other neuropsychiatric conditions such as schizophrenia.

## Conclusions

Pedigree analysis revealed autosomal recessive patterns in both families A and B. The disease was more prevalent in the children of consanguinity married couples in both families. Three genes, CDK5R1, OMG, and EV12A, on chromosome 17q11.2-q12 in the region of high LOD score showed increased expression in the frontal cortex of the brain, hippocampus, and spinal cord, respectively. Chromosomes 8, 9, and 11 also contained regions that contain NS-ID-relevant genes and can contribute to the disease. Distinguishing between S-ID and NS-ID is difficult because of the subtle syndromic features and needs proper diagnostic workup. Further research is needed to show the linkage of NS-ID genes with intelligence and other neuropsychiatric conditions.
